# Signatures of the topological *s*^+−^ superconducting order parameter in the type-II Weyl semimetal *T*_d_-MoTe_2_

**DOI:** 10.1038/s41467-017-01066-6

**Published:** 2017-10-20

**Authors:** Z. Guguchia, F. von Rohr, Z. Shermadini, A. T. Lee, S. Banerjee, A. R. Wieteska, C. A. Marianetti, B. A. Frandsen, H. Luetkens, Z. Gong, S. C. Cheung, C. Baines, A. Shengelaya, G. Taniashvili, A. N. Pasupathy, E. Morenzoni, S. J. L. Billinge, A. Amato, R. J. Cava, R. Khasanov, Y. J. Uemura

**Affiliations:** 1 Department of Physics, Columbia University, New York, NY 10027 USA; 2 Department of Chemistry, Princeton University, Princeton, NJ 08544 USA; 3 Laboratory for Muon Spin Spectroscopy, Paul Scherrer Institute, CH-5232 Villigen PSI, Switzerland; 4 Department of Applied Physics and Applied Mathematics, Columbia University, New York, NY 10027 USA; 5 Department of Physics, University of California, Berkeley, CA 94720 USA; 6 Department of Physics, Tbilisi State University, Chavchavadze 3, GE-0128 Tbilisi, Georgia; 7 Andronikashvili Institute of Physics of I. Javakhishvili Tbilisi State University, Tamarashvili Str. 6, 0177 Tbilisi, Georgia; 8 Condensed Matter Physics and Materials Science Department, Brookhaven National Laboratory, Upton, NY 11973 USA

## Abstract

In its orthorhombic *T*_d_ polymorph, MoTe_2_ is a type-II Weyl semimetal, where the Weyl fermions emerge at the boundary between electron and hole pockets. Non-saturating magnetoresistance and superconductivity were also observed in *T*_d_-MoTe_2_. Understanding the superconductivity in *T*_d_-MoTe_2_, which was proposed to be topologically non-trivial, is of eminent interest. Here, we report high-pressure muon-spin rotation experiments probing the temperature-dependent magnetic penetration depth in *T*_d_-MoTe_2_. A substantial increase of the superfluid density and a linear scaling with the superconducting critical temperature *T*_c_ is observed under pressure. Moreover, the superconducting order parameter in *T*_d_-MoTe_2_ is determined to have 2-gap *s*-wave symmetry. We also exclude time-reversal symmetry breaking in the superconducting state with zero-field μSR experiments. Considering the strong suppression of *T*_c_ in MoTe_2_ by disorder, we suggest that topologically non-trivial *s* ^+−^ state is more likely to be realized in MoTe_2_ than the topologically trivial *s* ^++^ state.

## Abstract (web summary)

Understanding the superconductivity in topological materials is of eminent interest. Here, Guguchia et al. report temperature-dependent magnetic penetration depth in the superconducting state of *T*_*d*_-MoTe_2_ under pressure, suggesting a topologically nontrivial *s* ^+−^ order parameter.

## Introduction

An interesting physical properties of two-dimensional materials such as transition metal dichalcogenides (TMDs) with a common formula, MX_2_ (M is a transition metal, X is a chalcogen atom), are useful for many emerging technological applications^1–19^. Depending on the crystal structure, TMDs can be either semiconducting or semimetallic^20–23^. The title compound MoTe_2_ undergoes a structural phase transition from monoclinic 1T′ to orthorhombic *T*_d_ at *T*_S_ ~ 250 K^15^. The 1T′ structure possesses the inversion symmetric space group *P*2_1_/*m*, whereas the *T*_d_ phase belongs to the non-centrosymmetric space group *Pmn*2_1_. Weyl fermions occur in the *T*_d_ phase where the inversion symmetry is broken and *T*_d_-MoTe_2_ is considered to be type-II Weyl semimetal^1, 2^. The evidence for the low temperature *T*_d_ structure in our MoTe_2_ sample is provided by X-ray pair distribution function (PDF) measurements (Supplementary Note 1; Supplementary Figs. 3 and 4). The Fermi surfaces in a type-II Weyl semimetal consist of a pair of electron pockets and hole pockets touching at the Weyl node, rather than at the point-like Fermi surface in traditional type-I WSM systems. Well fermions can arise by breaking either the space-inversion (SIS) or time-reversal symmetry (TRS)^24–26^. The different symmetry classifications of the Weyl semimetals are expected to exhibit distinct topological properties. Recent angle-resolved photoemission (ARPES) measurements^27^ and a high-field quantum oscillation study^28^ of the magnetoresistance (MR) in *T*_d_-MoTe_2_ revealed a distinctive features of surface states. In addition, in Mo_*x*_W_1−*x*_Te_2_, experimental signatures of the predicted topological connection between the Weyl bulk states and Fermi arc surface states were also reported^29^, constituting another unique property of Weyl semimetals.

*T*_d_-MoTe_2_ represents a rare example of a material with both superconductivity and a topologically non-trivial band structure. At ambient pressure, *T*_d_-MoTe_2_ is superconducting with *T*_c_ ≃ 0.1 K, but the application of a small pressure^15^ or the substitution of S for Te^30^ can markedly enhance *T*_c_. *T*_d_-MoTe_2_ is believed to be a promising candidate for topological superconductivity (TSC) in a bulk material. TSCs are materials with unique electronic states consisting of a full pairing gap in the bulk and gapless surface states composed of Majorana fermions (MFs)^24–26^. In general, topological superfluidity and superconductivity are well-established phenomena in condensed matter systems. The A-phase of superfluid helium-3 constitutes an example of a charge neutral topological superfluid, whereas Sr_2_RuO_4_^31^ is generally believed to be topological TRS-breaking superconductor. However, an example of a TRS invariant topological superconductor^24, 25^ is thus far unprecedented, and *T*_d_-MoTe_2_ may be a candidate material for this category. Until now, the only known properties of the superconducting state in *T*_d_-MoTe_2_ are the pressure-dependent critical temperatures and fields^15^. Thus, a thorough exploration of superconductivity in *T*_d_-MoTe_2_ from both experimental and theoretical perspectives is required.

To further explore superconductivity and its possible topological nature in *T*_d_-MoTe_2_, it is critical to measure the superconducting order parameter of *T*_d_-MoTe_2_ on the microscopic level through measurements of the bulk properties. Thus, we concentrate on high pressure^32–35^ muon-spin relaxation/rotation (μSR) measurements of the magnetic penetration depth *λ* in *T*_d_-MoTe_2_. This quantity is one of the fundamental parameters of a superconductor, as it is related to the superfluid density *n*_s_ via 1/*λ* ^2^ = *μ*_0_ *e* ^2^ *n*_s_/m* (where m* is the effective mass). Remarkably, the temperature dependence of *λ* is particularly sensitive to the topology of the SC gap: whereas in a nodeless superconductor, Δ*λ* ^−2^(*T*) ≡ *λ* ^−2^(0) − *λ* ^−2^(*T*) vanishes exponentially at low *T*, in a nodal SC it vanishes as a power of *T*. The μSR technique provides a powerful tool to measure *λ* in the vortex state of type-II superconductors in the bulk of the sample, in contrast to many techniques that probe *λ* only near the surface^36^. Details are provided in the “Methods” section. In addition, zero-field μSR has the ability to detect internal magnetic fields as small as 0.1 G without applying external magnetic fields, making it a highly valuable tool for probing spontaneous magnetic fields due to TRS breaking in exotic superconductors.

By combining high-pressure μSR and AC-susceptibility experiments, we observed a substantial increase of the superfluid density *n*_s_/m* and a linear scaling with *T*_c_ under pressure. Moreover, the superconducting order parameter in *T*_d_-MoTe_2_ is determined to have 2-gap *s*-wave symmetry. We also excluded time-reversal symmetry breaking in the high-pressure SC state, classifying MoTe_2_ as time-reversal-invariant superconductor with broken inversion symmetry. Taking into account the previous report on the strong suppression of *T*_c_ in MoTe_2_ by disorder, we suggest that topologically non-trivial *s* ^+−^ state is more likely to be realized in MoTe_2_ than the topologically trivial *s* ^++^ state. Should *s* ^+−^ indeed be the SC gap symmetry, the *T*_d_-MoTe_2_ is, to our knowledge, the first known example of a time-reversal-invariant topological (Weyl) superconductor.

## Results

### Probing the vortex state as a function of pressure

**Fig. 1:**
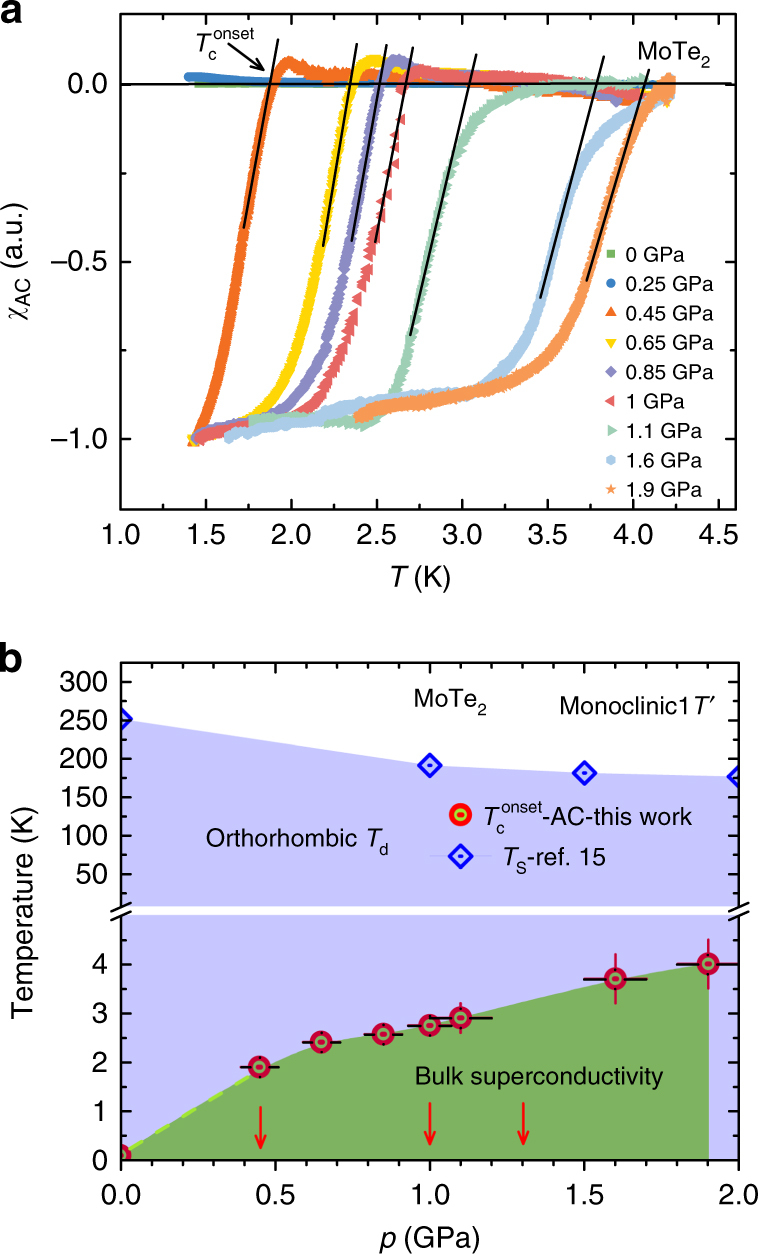
AC-susceptibility as a function of temperature and pressure in MoTe_2_. **a** Temperature dependence of the AC-susceptibility *χ*_AC_ for the polycrystalline sample of MoTe_2_, measured at ambient and various applied hydrostatic pressures up to *p* ≃ 1 GPa. The arrow denotes the superconducting transition temperature *T*_c_. **b** Pressure dependence of *T*_c_ (this work) and the structural phase transition temperature *T*_S_^15^. Arrows mark the pressures at which the *T*-dependence of the penetration depth was measured

Figure 1a shows the temperature dependence of the AC-susceptibility *χ*_AC_ of *T*_d_-MoTe_2_ in the temperature range between 1.4 and 4.2 K for selected hydrostatic pressures up to *p* = 1.9 GPa. A strong diamagnetic response and sharp SC transition are observed under pressure (Fig. 1), pointing to the high quality of the sample and providing evidence for bulk superconductivity in MoTe_2_^15^. The pressure dependence of *T*_c_ is shown in Fig. 1b. *T*_c_ increases with increasing pressure and reaches a critical temperature *T*_c_ ≃ 4 K at *p* = 1.9 GPa, the maximum applied pressure in the susceptibility experiments. The substantial increase of *T*_c_ from *T*_c_ ≃ 0.1 K at ambient pressure to *T*_c_ ≃ 4 K at moderate pressures in MoTe_2_ was considered as a manifestation of its topologically non-trivial electronic structure. Note that a strong pressure-induced enhancement of *T*_c_ has also been observed in topological superconductors such as Bi_2_Te_3_^37^ and Bi_2_Se_3_^38^. The temperature of the structural phase transition from monoclinic 1T′ to orthorhombic *T*_d_^15^ as a function of pressure is also shown in Fig. 1b. In the temperature and pressure range (*p* = 0–1.9 GPa) investigated here, MoTe_2_ is in the orthorhombic *T*_d_ structure. Moreover, density functional theory (DFT) calculations confirmed that in the pressure range investigated in this work, MoTe_2_ is a Weyl semimetal in which the band structure near the Fermi level is highly sensitive to changes in the lattice constants^15^.

**Fig. 2:**
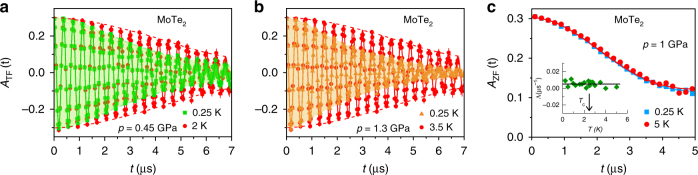
Transverse-field (TF) and zero-field (ZF) μSR-time spectra for MoTe_2_. The TF spectra are obtained above and below *T*_c_ in an applied magnetic field of *μ*_0_ *H* = 20 mT (after field cooling the sample from above *T*_c_) at *p* = 0.45 GPa (**a**) and *p* = 1.3 GPa (**b**). The solid lines in **a** and **b** represent fits to the data by means of Eq. (1). The dashed lines are guides to the eye. **c** ZF μSR time spectra for MoTe_2_ recorded above and below *T*_c_. The line represents the fit to the data with a Kubo–Toyabe depolarization function^39^, reflecting the field distribution at the muon site created by the nuclear moments. Error bars are the s.e.m. in about 10^6^ events. The error of each bin count *n* is given by the s.d. of *n*. The errors of each bin in *A*(*t*) are then calculated by s.e. propagation

Figure 2a and b displays the transverse-field (TF) μSR-time spectra for MoTe_2_ measured at *p* = 0.45 GPa and the maximum applied pressure *p* = 1.3 GPa, respectively, in an applied magnetic field of *μ*_0_ *H* = 20 mT. Spectra collected above the SC transition temperature (2 K, 3.5 K) and below it (0.25 K) are shown. The presence of the randomly oriented nuclear moments causes a weak relaxation of the μSR signal above *T*_c_. The relaxation rate is strongly enhanced below *T*_c_, which is caused by the formation of a flux-line lattice (FLL) in the SC state, giving rise to an inhomogeneous magnetic field distribution. Another reason for an enhancement of the relaxation rate could be magnetism, if present in the samples. However, precise zero-field (ZF)-μSR experiments does not show any indication of magnetism in *T*_d_-MoTe_2_ down to 0.25 K. This can be seen in ZF time spectra, shown in Fig. 2c, which can be well described only by considering the field distribution created by the nuclear moments^39^. Moreover, no change in ZF-μSR relaxation rate (see the inset of Fig. 2c) across *T*_c_ was observed, pointing to the absence of any spontaneous magnetic fields associated with a TRS^31, 40, 41^ breaking pairing state in MoTe_2_.

**Fig. 3:**
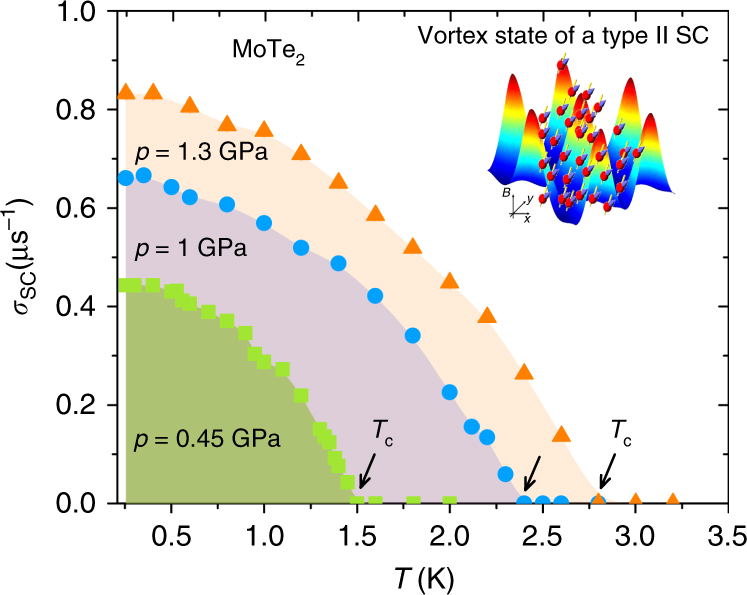
Superconducting muon-spin depolarization rate for MoTe_2_. The colored symbols represent the depolarization rate *σ*_sc_(*T*) measured in an applied magnetic field of *μ*_0_ *H* = 20 mT at various temperatures and hydrostatic pressures. The arrows mark the *T*_c_ values. Inset illustrates how muons, as local probes, sense the inhomogeneous field distribution in the vortex state of type-II superconductor. The error bars represent the s.d. of the fit parameters

Figure 3 displays the temperature dependence of the muon-spin depolarization rate *σ*_sc_ (measured in an applied magnetic field of *μ*_0_ *H* = 20 mT) in the SC state of MoTe_2_ at selected pressures. This relaxation rate is proportional to the width of the non-uniform field distribution (see “Methods” section). The formation of the vortex lattice below *T*_c_ causes an increase of the relaxation rate *σ*_sc_. As the pressure is increased, both the low-temperature value of *σ*_sc_(0.25 K) and the transition temperature *T*_c_ show a substantial increase (Fig. 3). *σ*_sc_(0.25 K) increases by a factor of ~2 from *p* = 0 GPa to *p* = 1.3 GPa. In the following, we show that the observed temperature dependence of *σ*_sc_, which reflects the topology of the SC gap, is consistent with the presence of the two isotropic *s*-wave gaps on the Fermi surface of MoTe_2_.

### Pressure-dependent magnetic penetration depth

To explore the symmetry of the SC gap, it is important to note that *λ*(*T*) is related to *σ*_sc_(*T*) as follows^42^:

1 $$\frac{{{\sigma _{{\rm{sc}}}}(T)}}{{{\gamma _{\rm{\mu }}}}} = 0.06091\frac{{{\Phi _0}}}{{\lambda _{{\rm{eff}}}^{\rm{2}}(T)}},$$

where Φ_0_ is the magnetic-flux quantum and *γ*_μ_ denotes the gyromagnetic ratio of the muon. Thus, the flat *T*-dependence of *σ*_sc_ at low temperature observed at various pressures (Fig. 3) implies an isotropic superconducting gap. In this case, $\lambda _{{\rm{eff}}}^{ - 2}\left( T \right)$ exponentially approaches its zero-temperature value. We note that it is the effective penetration depth *λ*_eff_ (powder average), which we extract from the μSR depolarization rate (Eq. (1)), and this is the one shown in the figures. In polycrystalline samples of highly anisotropic systems *λ*_eff_ is dominated by the shorter penetration depth *λ*_ab_ and *λ*_eff_ = 1.3*λ*_ab_ as previously shown^43, 44^.

The temperature dependence of the penetration depth is quantitatively described within the London approximation (*λ* ≫ *ξ*, where *ξ* is the coherence length) and by using the the empirical α-model. This model^45–49^ assumes, besides common *T*_c_, that the gaps in different bands are independent of each other. The superfluid densities, calculated for each component independently^49^, (see details in the “Methods” section) are added together with a weighting factor:

2 $$\frac{{{\lambda ^{ - 2}}(T)}}{{{\lambda ^{ - 2}}(0)}} = \alpha \frac{{\lambda _{{\rm{eff}}}^{ - 2}\left( {T,{\Delta _{0,1}}} \right)}}{{\lambda _{{\rm{eff}}}^{ - 2}\left( {0,{\Delta _{0,1}}} \right)}} + (1\!\! - \!\alpha )\frac{{\lambda _{{\rm{eff}}}^{ - 2}\left( {T,{\Delta _{0,2}}} \right)}}{{\lambda _{{\rm{eff}}}^{ - 2}\left( {0,{\Delta _{0,2}}} \right)}},$$

where *λ*_eff_(0) is the effective penetration depth at zero temperature, Δ_0,*i*_ is the value of the *i*-th SC gap (*i* = 1, 2) at *T* = 0 K, *α* and (1−*α*) are the weighting factors, which measure their relative contributions to *λ* ^−2^.

**Fig. 4:**
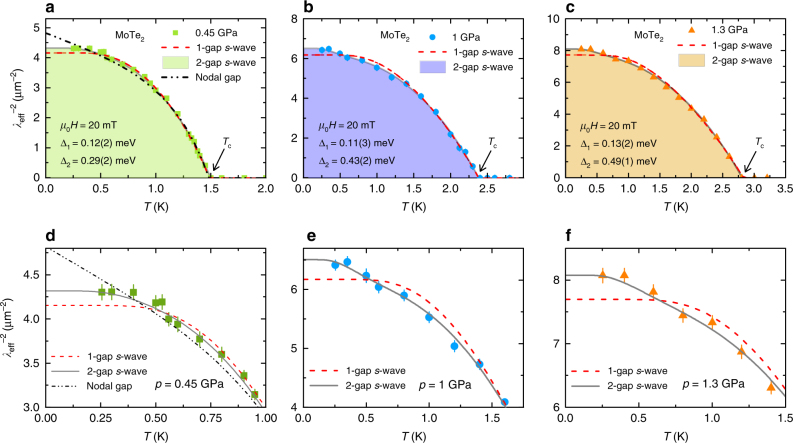
Pressure evolution of the penetration depth for MoTe_2_. Colored symbols represent the value of $\lambda _{{\rm{eff}}}^{ - 2}$ as a function of temperature, measured in an applied magnetic field of *μ*_0_ *H* = 20 mT under the applied hydrostatic pressures indicated in each panel. The solid lines correspond to a 2-gap *s*-wave model, the dashed and the dotted lines represent a fit using a 1-gap *s*-wave and nodal gap models, respectively. The error bars are calculated as the s.e.m

**Fig. 5:**
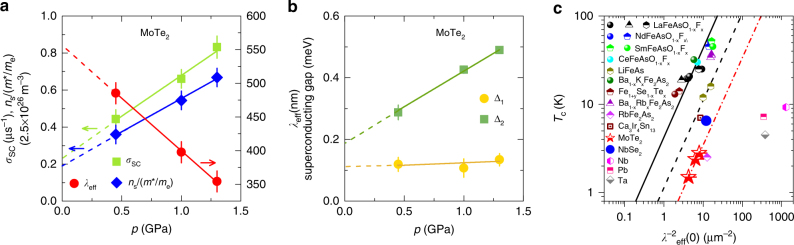
Pressure evolution of various quantities. The SC muon depolarization rate *σ*_SC_, magnetic penetration depth *λ*_eff_ and the superfluid density *n*_s_/m*m_e_ (**a**) as well as the zero-temperature gap values Δ_1,2_(0) (**b**) are shown as a function of hydrostatic pressure. Dashed lines are guides to the eye and solid lines represent linear fits to the data. The error bars represent the s.d. of the fit parameters. **c** A plot of *T*_c_ vs. $\lambda _{{\rm{eff}}}^{ - 2}(0)$ obtained from our μSR experiments in MoTe_2_. The dashed red line represents the linear fit to the MoTe_2_ data. The Uemura plot for various cuprate and Fe-based HTSs is also shown^49, 66–70^. The relation observed for underdoped cuprates is also shown (solid line for hole doping^55–59^ and dashed black line for electron doping^61^). The points for various conventional BCS superconductors and for NbSe_2_ are also shown

The results of this analysis are presented in Fig. 4a–f, where the temperature dependence of $\lambda _{{\rm{eff}}}^{ - 2}$ for MoTe_2_ is plotted at various pressures. We consider two different possibilities for the gap function: either a constant gap, Δ_0,*i*_ = Δ_*i*_, or an angle-dependent gap of the form Δ_0,*i*_ = Δ_*i*_ cos2*φ*, where *φ* is the polar angle around the Fermi surface. The dashed and the solid lines represent fits to the data using a 1-gap *s*-wave and a 2-gap *s*-wave model, respectively. The analysis appears to rule out the simple 1-gap *s*-wave model as an adequate description of $\lambda _{{\rm{eff}}}^{ - 2}$(*T*) for MoTe_2_. The 2-gap *s*-wave scenario with a small gap Δ_1_ ≃ 0.12(3) meV and a large gap Δ_2_ (with the pressure-independent weighting factor of 1−*α* = 0.87), describes the experimental data remarkably well. The possibility of a nodal gap was also tested, shown with a black dotted line in Fig. 4a, but was found to be inconsistent with the data. This conclusion is supported by a *χ* ^2^ test, revealing a value of *χ* ^2^ for the nodal gap model that is ~30% higher than the one for 2-gap *s*-wave model for *p* = 0.45 GPa. The ratios of the SC gap to *T*_c_ at *p* = 0.45 GPa were estimated to be 2Δ_1_/*k*_B_ *T*_c_ = 1.5(4) and 2Δ_2_/*k*_B_ *T*_c_ = 4.6(5) for the small and the large gaps, respectively. The ratio for the higher gap is consistent with the strong coupling limit BCS expectation^50^. However, a similar ratio can also be expected for Bose Einstein condensation (BEC)-like picture as pointed out in ref. ^51^. It is important to note that the ratio 2Δ/*k*_B_ *T*_c_ does not effectively distinguish between BCS or BEC. This is particularly true in two band systems, where the ratio is not universal even in the BCS limit, as it depends also on the density of states of the two bands. The pressure dependence of various physical parameters are plotted in Fig. 5a and b. From Fig. 5a, a substantial decrease of *λ*_eff_(0) (increase of *σ*_sc_) with pressure is evident. At the highest applied pressure of *p* = 1.3 GPa, the reduction of *λ*_eff_(0) is ~25% compared with the value at *p* = 0.45 GPa. The small gap Δ_1_ ≃ 0.12(3) meV stays nearly unchanged by pressure, whereas the large gap Δ_2_ increases from Δ_2_ ≃ 0.29(1) meV at *p* = 0.45 GPa to Δ_2_ ≃ 0.49(1) meV at *p* = 1.3 GPa, i.e., by ~70%.

In general, the penetration depth *λ* is given as a function of *n*_s_, m*, *ξ*, and the mean free path *l* as

3 $$\begin{array}{*{20}{l}}\\ {\frac{1}{{{\lambda ^2}}} = \frac{{4\pi {n_{\rm{s}}}{e^2}}}{{{m^*}{c^2}}} \times \frac{1}{{1 + \xi {\rm{/}}l}}.} \hfill \\ \end{array}$$

For systems close to the clean limit, *ξ*/*l* → 0, the second term essentially becomes unity, and the simple relation 1/*λ* ∝ *n*_s_/m* holds. Considering the *H*_c2_ values of MoTe_2_ reported in ref. ^15^, we estimated *ξ* ≃ 26 and 14 nm for *p* = 0.45 and 1 GPa, respectively. At ambient pressure, the in-plane mean free path *l* was estimated to be *l* ≃ 100–200 nm^28^. No estimates are currently available for *l* under pressure. However, in-plane *l* is most probably independent of pressure, considering the fact that the effect of compression is mostly between layers rather than within layers, thanks to the unique anisotropy of the van der Waals structure. In particular, the intralayer Mo–Te bond length is almost unchanged by pressure, especially in the pressure region relevant to this study. Thus, in view of the short coherence length and relatively large *l*, we can assume that MoTe_2_ lies close to the clean limit^52^. With this assumption, we obtain the ground-state value *n*_s_/(m*/*m*_e_) ≃ 0.9 × 10^26^ m^−3^, 1.36 × 10^26^ m^−3^, and 1.67 × 10^26^ m^−3^ for *p* = 0.45, 1, and 1.3 GPa respectively. Interestingly, *n*_s_/(m*/*m*_e_) increases substantially under pressure, which will be discussed below.

## Discussion

One of the essential findings of this paper is the observation of two-gap superconductivity in *T*_d_-MoTe_2_. Recent ARPES^27^ experiments on MoTe_2_ revealed the presence of three bulk hole pockets (a circular hole pocket around the Brillouin zone center and two butterfly-like hole pockets) and two bulk electron pockets, which are symmetrically distributed along the Γ-X direction with respect to the Brillouin zone center Γ. As several bands cross the Fermi surface in MoTe_2_, two-gap superconductivity can be understood by assuming that the SC gaps open at two distinct types of bands. Now the interesting question arises: How consistent is the observed two-gap superconductivity with the possible topological nature of superconductivity in *T*_d_-MoTe_2_? Note that the superconductor *T*_d_-MoTe_2_ represents a time-reversal-invariant Weyl semimetal, which has broken inversion symmetry. Recently, the detailed studies of microscopic interactions and the SC gap symmetry for time-reversal-invariant TSC in Weyl semimetals were performed^24^. Namely, it was shown that for TSC the gaps can be momentum independent on each FS but must change the sign between different FSs. μSR experiments alone cannot distinguish between sing-changing *s* ^+−^ (topological) and *s* ^++^ (trivial) pairing states. However, considering the recent experimental observations of the strong suppression of *T*_c_ in MoTe_2_ by disorder^11, 53^ and the theoretical proposal that TSC is more sensitive to disorder than the ordinary *s*-wave superconductivity^24, 54^, we suggest that *s* ^+−^ state is more likely to be realized than the trivial *s* ^++^ state. Further phase sensitive experiments are desirable to distinguish between *s* ^+−^ and *s* ^++^ states in MoTe_2_.

**Fig. 6:**
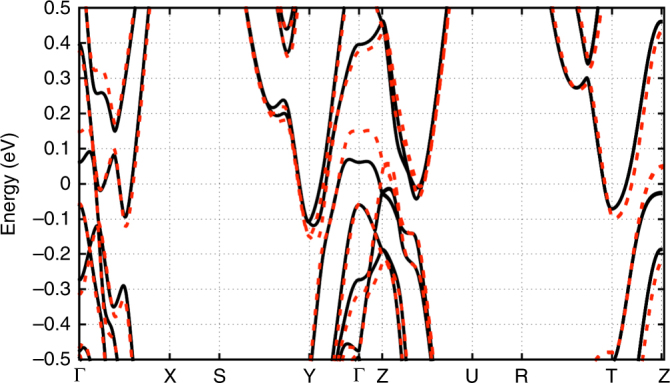
DFT results. Calculated band structure of *T*_d_-MoTe_2_ at ambient *p* (solid black curves) and for *p* = 1.3 GPa (dashed red curves)

Besides the two-gap superconductivity, another interesting observation is the strong enhancement of the superfluid density $\lambda _{{\rm{eff}}}^{ - 2}(0)$ ∝ *n*_s_/(m*/*m*_e_) and its linear scaling with *T*_c_ (Fig. 5c). Between *p* = 0.45 and 1.3 GPa, *n*_s_/(m*/*m*_e_) increases by factor of ~1.8. We also compared the band structures for ambient as well as for the hydrostatic pressure of 1.3 GPa by means of DFT calculations. The results are shown in Fig. 6. When the pressure is applied, there are appreciable differences of the bands near the Fermi level, especially near *Y* − *Z*, *T* − *Z*, and Γ − *X*. Near Γ, the hole band is shifted by +0.8–0.9 eV, whereas the electron band at *Y* and *T* are lowered by 20–40 meV.

The nearly linear relationship between *T*_c_ and the superfluid density was first noticed in hole-doped cuprates in 1988–1989^55, 56^, and its possible relevance to the crossover from BEC to BCS condensation has been discussed in several subsequent papers^57–59^. The linear relationship was noticed mainly in systems lying along the line for which the ratio of *T*_c_ to the effective Fermi temperature *T*_F_ is about *T*_c_/*T*_F_ ~ 0.05, implying a reduction of *T*_c_ by a factor of 4–5 from the ideal Bose condensation temperature for a non-interacting Bose gas composed of the same number of Fermions pairing without changing their effective masses. The present results on MoTe_2_ and NbSe_2_^60^ in Fig. 5c demonstrate that a linear relation holds for these systems, but with the ratio *T*_c_/*T*_F_ being reduced by a factor of 16–20. It was also noticed^61^ that electron-doped cuprates follow another line with their *T*_c_/*T*_F_ reduced by a factor of ~4 from the line of hole-doped cuprates. As the present system MoTe_2_ and NbSe_2_ fall into the clean limit, the linear relation is unrelated to pair breaking, and can be expected to hold between *T*_c_ and *n*_s_/m*.

In a naive picture of BEC to BCS crossover, systems with small *T*_c_/*T*_F_ (large *T*_F_) are considered to be on the “BCS” side, whereas the linear relationship between *T*_c_ and *T*_F_ is expected only on the BEC side. Figure 5c indicates that the BEC-like linear relationship may exist in systems with *T*_c_/*T*_F_ reduced by a factor 4 to 20 from the ratio in hole-doped cuprates, presenting a new challenge for theoretical explanations.

In conclusion, we provide the first microscopic investigation of the superconductivity in *T*_d_-MoTe_2_. Specifically, the zero-temperature magnetic penetration depth *λ*_eff_(0) and the temperature dependence of $\lambda _{{\rm{eff}}}^{ - 2}$ were studied in the type-II Weyl semimetal *T*_d_-MoTe_2_ by means of μSR experiments as a function of pressure up to *p* ≃ 1.3 GPa. Remarkably, the temperature dependence of $1{\rm{/}}\lambda _{{\rm{eff}}}^2\left( T \right)$ is inconsistent with a simple isotropic *s*-wave pairing symmetry and with presence of nodes in the gap. However, it is well described by a 2-gap *s*-wave scenario, indicating multigap superconductivity in MoTe_2_. We also excluded time-reversal symmetry breaking in the high-pressure SC state with sensitive zero-field μSR experiments, classifying MoTe_2_ as time-reversal-invariant superconductor with broken inversion symmetry. In this type of superconductor, a 2-gap *s*-wave model is consistent with a topologically non-trivial superconducting state if the gaps Δ_1_ and Δ_2_ existing on different Fermi surfaces have opposite signs. μSR experiments alone cannot distinguish between sign changing *s* ^+−^ (topological) and *s* ^++^ (trivial) pairing states. However, considering the previous report on the strong suppression of *T*_c_ in MoTe_2_ by disorder, we suggest that *s* ^+−^ state is more likely to be realized in MoTe_2_ than the *s* ^++^ state. Should *s* ^+−^ be the SC gap symmetry, the high-pressure state of MoTe_2_ is, to our knowledge, the first known example of a Weyl superconductor, as well as the first example of a time-reversal invariant topological (Weyl) superconductor. Finally, we observed a linear correlation between *T*_*c*_ and the zero-temperature superfluid density $\lambda _{{\rm{eff}}}^{ - 2}(0)$ in MoTe_2_, which together with the observed two-gap behavior, points to the unconventional nature of superconductivity in *T*_d_-MoTe_2_. We hope the present results will stimulate theoretical investigations to obtain a microscopic understanding of the relation between superconductivity and the topologically non-trivial electronic structure of *T*_d_-MoTe_2_.

## Methods

### Sample preparation

High quality single crystals and polycrystalline samples were obtained by mixing of molybdenum foil (99.95%) and tellurium lumps (99.999+%) in a ratio of 1:20 in a quartz tube and sealed under vacuum. The reagents were heated to 1000 °C within 10 h. They dwelled at this temperature for 24 h, before they were cooled to 900 °C within 30 h (polycrystalline sample) or 100 h (single crystals). At 900 °C the tellurium flux was spined-off and the samples were quenched in air. The obtained MoTe_2_ samples were annealed at 400 °C for 12 h to remove any residual tellurium.

### Pressure cell

Single wall CuBe piston-cylinder type of pressure cell is used together with Daphne oil to generate hydrostatic pressures for μSR experiments^32, 33^. Pressure dependence of the SC critical temperature of tiny indium piece is used to measure the pressure. The fraction of the muons stopping in the sample was estimated to be ~40%.

### μSR experiment

Nearly perfectly spin-polarized, positively charged muons *μ* ^+^ are implanted into the specimen, where they behave as very sensitive microscopic magnetic probes. Muon-spin experiences the Larmor precession either in the local field or in an applied magnetic field. Fundamental parameters such as the magnetic penetration depth *λ* and the coherence length *ξ* can be measured in the bulk of a superconductor by means of transverse-field μSR technique, in which the magnetic field is applied perpendicular to the initial muon-spin polarization. If a type-II superconductor is cooled below *T*_c_ in an applied magnetic field ranged between the lower (*H*_c1_) and the upper (*H*_c2_) critical fields, a flux-line lattice is formed and muons will randomly probe the non-uniform field distribution of the vortex lattice.

Combination of high-pressure μSR instrument GPD (*μ*E1 beamline), the low-background instrument GPS (*π*M3 beamline) and the low-temperature instrument LTF (*π*M3.3) of the Paul Scherrer Institute (Villigen, Switzerland) is used to study the single crystalline as well as the polycrystalline samples of MoTe_2_.

### Analysis of TF-μSR data

The following function is used to analyze the TF μSR data^45^:

4 $$\begin{array}{*{20}{l}}\\ {P(t)} \hfill & = \hfill & {{A_{\rm{s}}}\,{\rm{exp}}\left[ { - \frac{{\left( {\sigma _{{\rm{sc}}}^2 + \sigma _{{\rm{nm}}}^{\rm{2}}} \right){t^2}}}{2}} \right]{\rm{cos}}\left( {{\gamma _{\rm{\mu }}}{B_{{\rm{int}},{\rm{s}}}}t + \varphi } \right)} \hfill \\ {} \hfill & {} \hfill & { + {A_{{\rm{pc}}}}\,{\rm{exp}}\left[ { - \frac{{\sigma _{{\rm{pc}}}^{\rm{2}}{t^2}}}{2}} \right]{\rm{cos}}\left( {{\gamma _{\rm{\mu }}}{B_{{\rm{int}},{\rm{pc}}}}t + \varphi } \right)} \hfill \\ \end{array}.$$

Here *A*_s_ and *A*_pc_ denote the initial assymmetries of the sample and the pressure cell, respectively. $\gamma {\rm{/}}(2\pi ) \simeq 135.5$ MHz/*T* is the gyromagnetic ratio of muon and *φ* denotes the initial phase of the muon-spin ensemble. *B*_int_ represents the internal magnetic field, sensed by the muons. *σ*_nm_ is the relaxation rate, caused by the nuclear magnetic moments. The value of *σ*_nm_ was obtained above *T*_c_ and was kept constant over the entire temperature range. The relaxation rate *σ*_sc_ describes the damping of the μSR signal due to the formation of the vortex lattice in the SC state. *σ*_pc_ describes the depolarization due to the nuclear moments of the pressure cell. *σ*_pc_ exhibits the temperature dependence below *T*_c_ due to the influence of the diamagnetic moment of the SC sample on the pressure cell^34^. The linear coupling between *σ*_pc_ and the field shift of the internal magnetic field in the SC state was assumed to consider the temperature-dependent *σ*_pc_ below *T*_c_: *σ*_pc_(*T*) = *σ*_pc_(*T* > *T*_c_) + *C*(*T*)(*μ*_0_ *H*_int,NS_ − *μ*_0_ *H*_int,SC_), where *σ*_pc_(*T* > *T*_c_) = 0.25 μs^−1^ is the temperature-independent Gaussian relaxation rate. *μ*_0_ *H*_int,NS_ and *μ*_0_ *H*_int,SC_ are the internal magnetic fields measured in the normal and in the SC state, respectively. As demonstrated by the solid lines in Fig. 2b and c, the μSR data are well described by Eq. (1).

### Analysis of *λ*(*T*)

*λ*_eff_(*T*) was calculated by considering the London approximation (*λ* ≫ *ξ*) using the following function^45, 46^:

5 $$\frac{{\lambda _{{\rm{eff}}}^{ - 2}\left( {T,{\Delta _{0,i}}} \right)}}{{\lambda _{{\rm{eff}}}^{ - 2}\left( {0,{\Delta _{0,i}}} \right)}} = 1 + \frac{1}{\pi }{\int}_{\!\!\!\!\!0}^{2\pi } {\int}_{\!\!\!\!\!{\Delta _{\left( {T,\varphi } \right)}}}^\infty \left( {\frac{{\partial f}}{{\partial E}}} \right)\frac{{E{\rm{d}}E{\rm{d}}\varphi }}{{\sqrt {{E^2} \!\!- \!\!{\Delta _i}{{\left( {T,\varphi } \right)}^2}} }},$$

where *f* = [1 + exp(*E*/*k*_B_ *T*)]^−1^ represents the Fermi function, *φ* is the angle along the Fermi surface, and Δ_*i*_(*T*, *φ*) = Δ_0,*i*_Γ(*T*/*T*_c_)*g*(*φ*) (Δ_0,*i*_ is the maximum gap value at *T* = 0). The temperature evolution of the gap is given by the expression Γ(*T*/*T*_c_) = tanh{1.82[1.018(*T*_c_/*T* − 1)]^0.51^}^47^, whereas *g*(*φ*) takes care of the angular dependence of the superconducting gap. Namely, *g*(*φ*) = 1 in the case of both a 1-gap *s*-wave and a 2-gap *s*-wave, and |cos(2*φ*)| for a nodal gap.

### DFT calculations of the electronic band structure

We used van der Waals density (vdW) functional and the projector-augmented wave (PAW) method^62^, as implemented in the VASP code^63^. We adopted the generalized gradient approximation (GGA) proposed by Perdew et al. (PBE)^64^ and DFT-D2 vdW functional proposed by Grimme et al.^65^ as a nonlocal correlation. Spin–orbit coupling (SOC) is included in all cases. A plane wave basis with a kinetic energy cutoff of 500 eV was employed. We used a Γ-centered **k**-point mesh of 15 × 9 × 5. Optimized lattice parameters of *T*_d_ phase are *a* = 3.507, *b* = 6.371, and *c* = 13.743 Å, close to the previous experimental values; (*a*, *b*, *c*) = (3.468, 6.310, 13.861)^8^ and (3.458, 6.304, 13.859)^3^.

### Data availability

All relevant data are available from the authors. The data can also be found at the following link http://musruser.psi.ch/cgi-bin/SearchDB.cgi using the details: GPD, Year: 2016, Run Title: MoTe_2_.

## Electronic supplementary material

Supplementary Information

Peer Review File
