# Author Correction: Signatures of the topological *s*^+−^ superconducting order parameter in the type-II Weyl semimetal *T*_*d*_-MoTe_2_

**DOI:** 10.1038/s41467-017-02460-w

**Published:** 2018-01-10

**Authors:** Z. Guguchia, F. von Rohr, Z. Shermadini, A. T. Lee, S. Banerjee, A. R. Wieteska, C. A. Marianetti, B. A. Frandsen, H. Luetkens, Z. Gong, S. C. Cheung, C. Baines, A. Shengelaya, G. Taniashvili, A. N. Pasupathy, E. Morenzoni, S. J. L. Billinge, A. Amato, R. J. Cava, R. Khasanov, Y. J. Uemura

**Affiliations:** 1 Department of Physics, Columbia University, New York, NY 10027 USA; 2 Department of Chemistry, Princeton University, Princeton, NJ 08544 USA; 3 Laboratory for Muon Spin Spectroscopy, Paul Scherrer Institute, CH-5232 Villigen PSI, Switzerland; 4 Department of Applied Physics and Applied Mathematics, Columbia University, New York, NY 10027 USA; 5 Department of Physics, University of California, Berkeley, CA 94720 USA; 6 Department of Physics, Tbilisi State University, Chavchavadze 3, GE-0128 Tbilisi, Georgia; 7 Andronikashvili Institute of Physics of I. Javakhishvili Tbilisi State University, Tamarashvili str. 6, 0177 Tbilisi, Georgia; 8 Condensed Matter Physics and Materials Science Department, Brookhaven National Laboratory, Upton, NY 11973 USA

*Nature Communications* 10.1038/s41467-017-01066-6, Article published online 20 October 2017

The original version of this article omitted the following from the Acknowledgements: “CAM and AL were supported by the NSF MRSEC program through Columbia in the Center for Precision Assembly of Superstratic and Superatomic Solids (DMR-1420634). Additionally, this research used resources of the National Energy Research Scientific Computing Center, a DOE Office of Science User Facility supported by the Office of Science of the U.S. Department of Energy under ‘Contract No. DE-AC02-05CH11231’.” This has now been corrected in both the PDF and HTML versions of the article.

