# Supplementary material for: Signatures of the topological s+− superconducting order parameter in the type-II Weyl semimetal Td-MoTe2
Source: Nat Commun. 2017 Oct 20;8:1082. doi: 10.1038/s41467-017-01066-6 (PMC5651900; doi:10.1038/s41467-017-01066-6)
Supplement: Supplementary file 1 — Supplementary Information [file 41467_2017_1066_MOESM1_ESM.pdf]

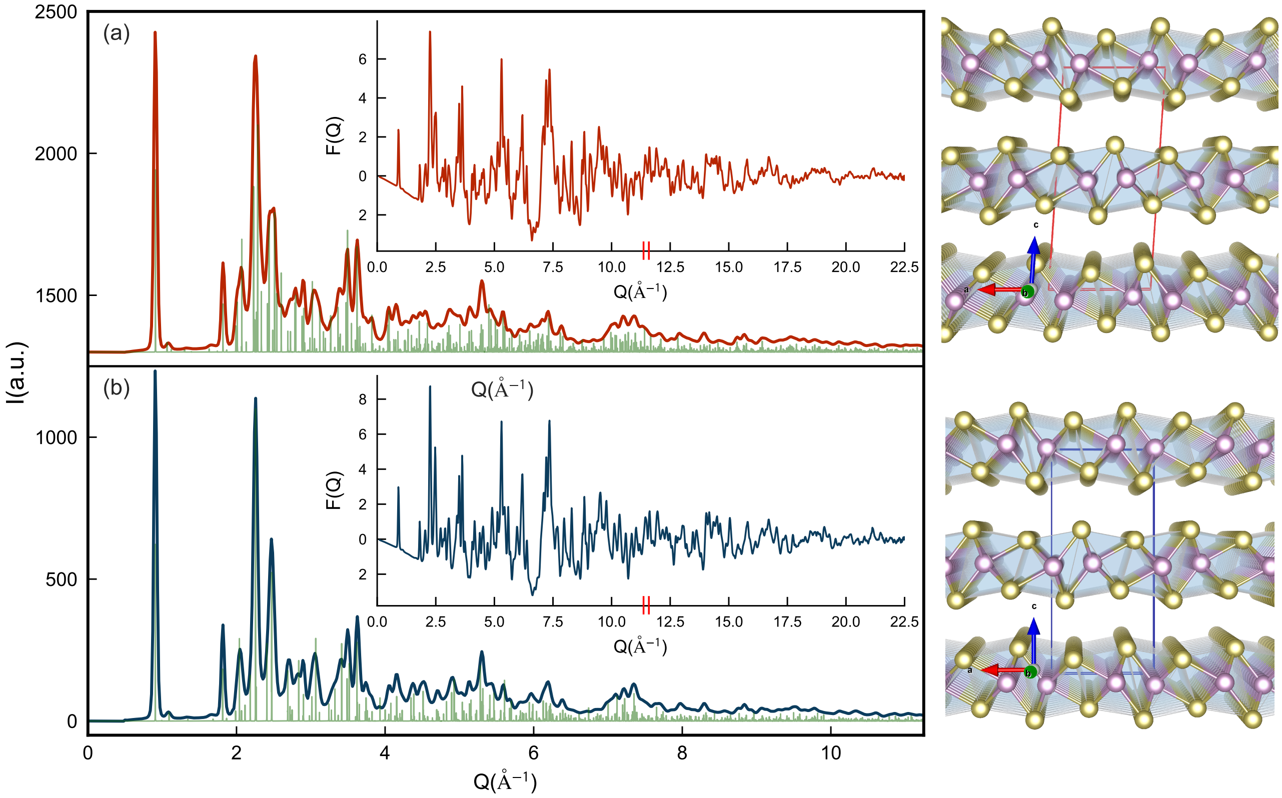

Supplementary Figure 1. **Powder diffraction patterns of $MoTe_2$.** Patterns recorded at 300K (a) and 100K (b) plotted as a function of Q and truncated where Bragg peaks are no longer distinguishable. The insets show the total scattering structure function $F(Q)$ where the full range used for the PDF transformation is provided. Small vertical lines in red mark the upper limit of $I(Q)$ given in (a,b), past which a significant amount of scattered intensity is seen in the structure function. Fine green lines correspond to calculated Bragg peaks from candidate models T' (a) and T_d (b). To the right we show the centrosymmetric monoclinic structure plotted above the non-centrosymmetric orthorhombic structure, from references discussed in the text. The $MoTe_6$ octahedral units are shaded in blue. Note that in order to provide a useful projection to compare T' and T_d, the unit cell orientation for the orthorhombic structure is changed from the standard (abc) setting for $Pmn2_1$ to (ba-c) for the symmetry equivalent $Pnm2_1$ space group.

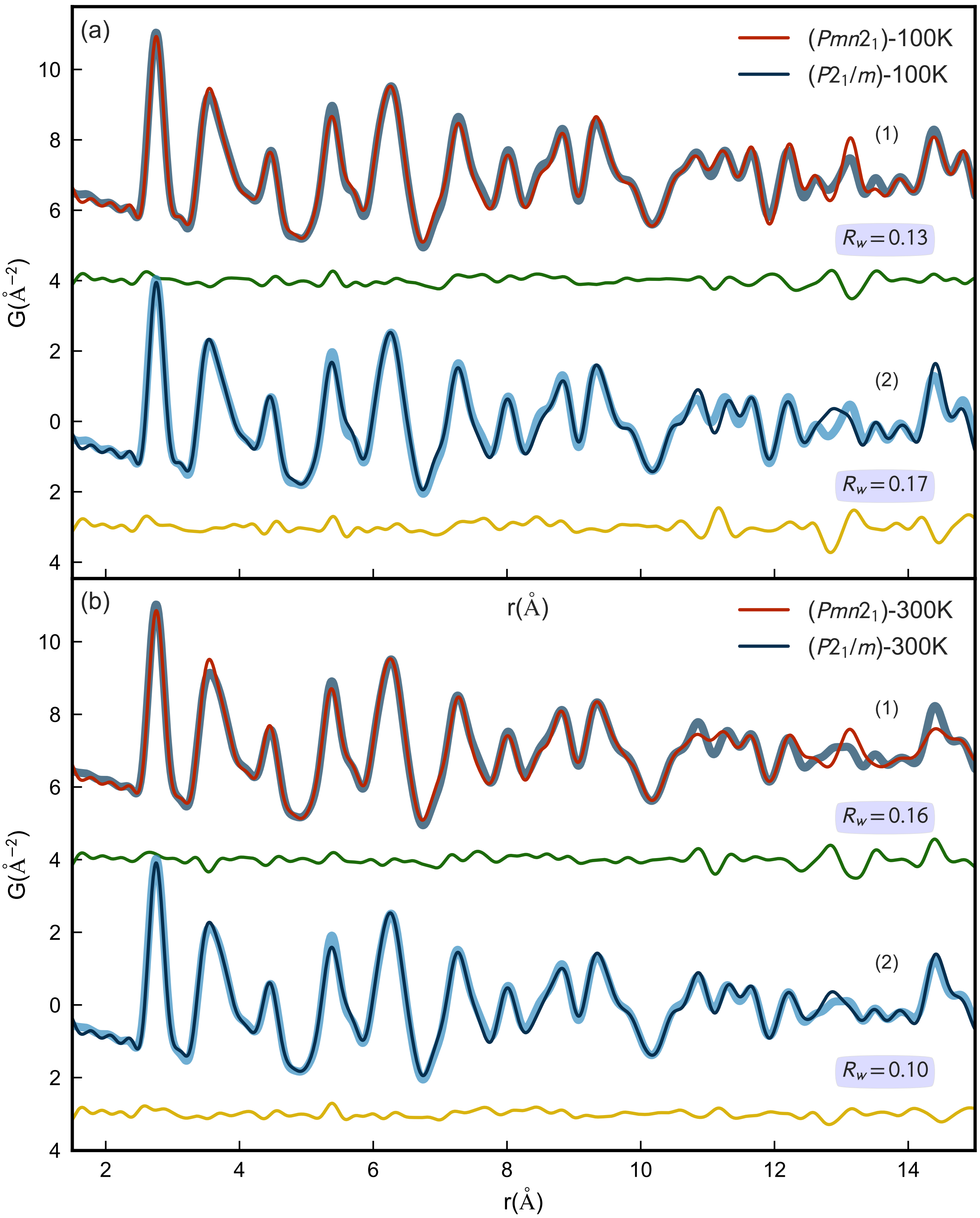

Supplementary Figure 2. **Pair Distribution Function Analysis of $MoTe_2$ data.** (a) PDF local structure refinements of $MoTe_2$ measured at 100K fit to the T_d structure (1) and the T' structure (2). (b) an analogous comparison for the 300K measurement. Thick curves are the raw PDF data, and the calculated PDF from model is overlayed as thin solid lines, and labeled in the legend. Offset difference curves in green and yellow are simply the calculated PDF subtracted from the measured PDF. The goodness-of-fit parameter (R_w) is provided for each of the four refinements, which confirm that the 100 K data is better described as T_d and the 300K data as T'.

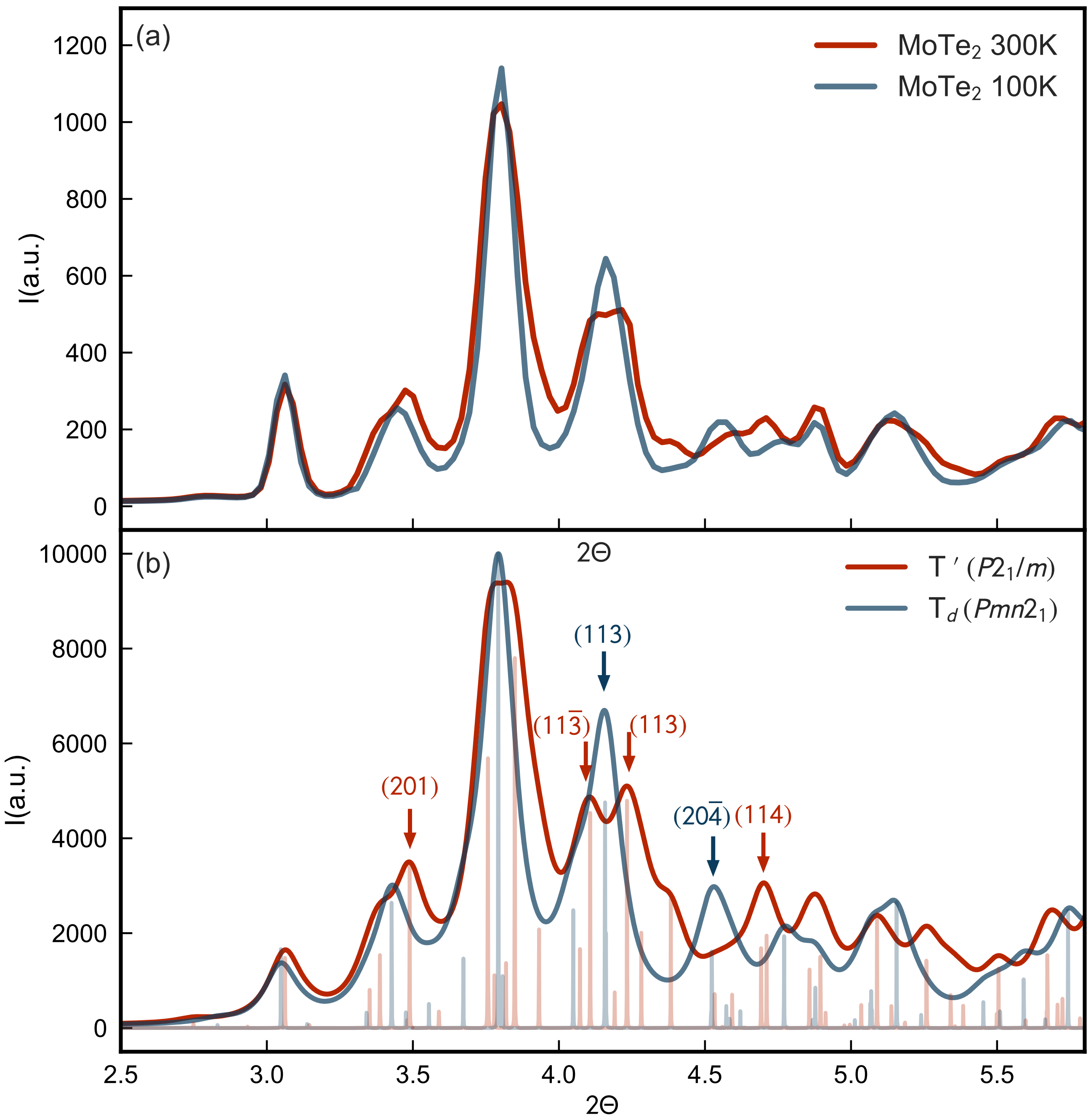

Supplementary Figure 3. **Comparison of high and low-T diffraction patterns.** (a) Measured diffraction patterns at 300K and 100K using an incident wavelength of $\lambda = 0.18470$ Å. (b) Bragg profile calculated from unmodified candidate structures. Solid curves in red and blue are the Bragg peaks calculated from the reflections (sharp lines), broadened uniformly with an FWHM=0.1 in 2θ (=0.059Å^{-1} in Q). The orthorhombic (113) reflection measured at 100K, splits into the $(11\bar{3})$ and (113) at 300K, which was also reported for WTe_2 [1]

I. SUPPLEMENTARY NOTE 1: CONFIRMATION OF THE ORTHORHOMBIC (T_d) TO MONOCLINIC ($T^{'}$) PHASE TRANSITION IN MOTE$_2$

Total scattering measurements were carried out on the XPD (28-ID-2) beamline at the National Synchrotron Light Source II (NSLS-II), Brookhaven National Laboratory. A finely ground powder of $MoTe_2$ was prepared in an inert Argon chamber, and sealed in 1.02mm (OD) polyimide capillaries. Diffraction patterns were collected in a Debye-Scherrer geometry with an X-ray energy of 67.127 keV ($\lambda = 0.18470$ Å) using a large-area 2D PerkinElmer detector (2048^2 pixels with 200 μm^2 pixel size). The detector was mounted with a sample-to-detector distance of 344.79 mm, to achieve a balance between q-resolution and q-range. The sample was measured at 100K and 300K using an Oxford CS-700 cryostream, allowing ample time for the material to thermalize. The experimental geometry, 2θ range, and detector misorientations were calibrated by measuring a crystalline nickel powder directly

prior to data collection at each temperature point, with the experimental geometry parameters refined using the PyFAI program [2].

Raw 2D diffraction patterns are azimuthally integrated to obtain the 1D scattering intensity $I(Q)$ where $Q = 4\pi \sin\theta/\lambda$. Standardized corrections are then made to the data and $I(Q)$ is normalized by dividing by the total scattering cross-section of the sample, resulting in the structure function $S(Q)$

$$S(Q) - 1 = \frac{I_\mathrm{c}(Q) - \sum c_\mathrm{i} \left| f_\mathrm{i}(Q)^2 \right|}{\sum c_\mathrm{i} \left| f_\mathrm{i}(Q)^2 \right|} \tag{1}$$

where I_c is the coherent scattering intensity as a function of the momentum transfer Q and c_i and f_i are the concentration and X-ray scattering factor, per atom type i. Because the scattering cross section becomes very small at high-Q, an important result of this normalization is that the high angle data are significantly amplified [3]. This is illustrated in Figure 1(a,b) where we show the standard integrated XRD patterns $I(Q)$ versus the reduced total scattering structure function $F(Q)$ (insets), where $F(Q) = Q[S(Q) - 1]$. While the raw diffraction pattern appears to have almost no distinguishable peaks near the upper limit of the shortened Q-range, the form factor normalized $F(Q)$ contains well resolved, high amplitude peaks extending to a Q-range twice the maximum value shown for $I(Q)$. The high degree of structural coherence observed for $MoTe_2$ is in contrast to diffraction studies of similar layered TMDs, where diffuse scattering from turbostratic disorder and other defects often dominates the high angle signal[4].

The full Q-range where structure can be resolved, including both Bragg and diffuse components, is shown in the insets of Figure 1. The structure function is Fourier transformed to obtain the pair distribution function (PDF), using PDFgetX3 [5] within xPDFsuite [6]. The experimental PDF, $G(r)$, is the truncated Fourier transform of $F(Q)$

$$G(r) = \frac{2}{\pi} \int_{Q_\mathrm{min}}^{Q_\mathrm{max}} F(Q) \sin(Qr)\, \mathrm{d}Q, \tag{2}$$

For both $MoTe_2$ measurements (100K and 300K), the Q_max was chosen to be 22.5 Å^{-1} to give the best tradeoff between statistical noise and real-space resolution.

The PDF is a histogram of interatomic distances that gives the probability of finding pairs of atoms in a material, separated by a distance r. $G(r)$ can be calculated from a structure model according to

$$G(r) = 4\pi r \left[\rho(r) - \rho_0\right], \tag{3}$$
$$\rho(r) = \frac{1}{4\pi r^2 N} \sum_i \sum_{j \neq i} \frac{f_i f_j}{\langle f \rangle^2} \delta(r - r_{ij}).$$

Here, ρ_0 is the atomic number density of the material and $\rho(r)$ is the atomic pair density, which is the mean weighted density of neighbor atoms at distance r from an atom at the origin. The sums in $\rho(r)$ run over all atoms (N) in the model, with periodic boundary conditions. f_i is the scattering factor of atom i, $\langle f \rangle$ is the average scattering factor and r_{ij} is the distance between atoms i and j. Supplementary Eqs. 3 are used to fit the PDF calculated from a model to the experimentally measured PDFs. The PDFgui program was used to construct unit cells from reference structures, carry out the refinements, and determine the agreement between simulated PDFs and data, quantified by the residual function R_w [7].

We included three candidate structure models for local structure refinements of $MoTe_2$ PDFs measured at 100K and 300K. The monoclinic 1T′-$MoTe_2$ (SG: $P2_1/m$) structure reported by B.E. Brown [8], the orthorhombic $1T_d$-$MoTe_2$ (SG: $Pmn2_1$) structure, also referred to as the γ form, and a hexagonal 2H polytype (SG:$P6_3/mmc$) reported by D. Puotinen and R.E. Newnham [9]. The 2H structure was ruled out as both 1T structures gave significantly better fits for both low-temperature and high-temperature datasets. The T′ and T_d PDF refinements were performed conservatively by adjusting just a few parameters in the structure models: lattice (a,b,c), scale, delta-2 (a parameter for correlated motion effects), one isotropic atomic displacement parameter (ADP) for the 4 Mo atoms, and one ADP for the 8 Te atoms, with both structures containing 12 atoms in the unit cell. For the monoclinic phase, an additional symmetry allowed β angle was refined. Atomic positions were not refined. The instrumental resolution parameters $Q_\mathrm{damp} = 0.0217$ and $Q_\mathrm{broad} = 0.009$ were determined through refinements of the nickel calibrant, and kept fixed for all other refinements.

PDFs for $MoTe_2$ are refined over an r-range from $1.5 < r < 15$ Å, slightly larger than the c-axis length for both T′ and T_d models. The PDF at low-r contains structural information from the full $F(Q)$ range and as a result, refinements are very sensitive to deviations from the average structure. This is advantageous when characterizing the local atomic environment for $MoTe_2$, with both candidate models containing layers of edge-sharing, distorted $MoTe_6$ octahedra (Figure 1) within the refinement range selected. The approach is different from traditional powder XRD Rietveld refinements of similar materials, where a substantial amount of diffuse scattering is neglected, and phase identification is restricted to a small number of low angle Bragg reflections, thus increasing the number of possible structures that may fit equivalently to the same data.

The results of the PDF analysis are summarized in Figure 2. For $MoTe_2$ measured at 100K there is a clear

improvement in the agreement factor (R_{w}) when the data is refined to the orthorhombic T_{d} model, with smaller residuals indicating better fits. The difference in refinement quality is more significant above the orthorhombic to monoclinic phase transition ($T_{\mathrm{s}} \sim 250K$) and the 300K PDF is in good agreement with the T' model. By varying a small number of structural parameters we ensure that these differences in R_{w} accurately represent a structural transformation. Additionally, the refined lattice constants differ minimally from the starting values provided in the reference T' and T_{d} models. The best-fit PDF at 100K using the T_{d} structure, yields refined lattice parameters of $a = 3.474$Å, $b = 6.328$Å, $c = 13.884$Å, and small ADPs, U_{iso}(Mo)= 0.0043; U_{iso}(Te)= 0.0056. For $MoTe_2$ at 300K, the monoclinic structure refines $a = 6.335$Å, $b = 3.471$Å, $c = 13.853$Å; $\beta = 93.886°$ and U_{iso}(Mo)= 0.0058; U_{iso}(Te)= 0.0064.

In Figure 3, we index the Bragg profile to support the results from the more quantitative PDF refinements. Here we simply simply compare the raw diffraction patterns measured at 100K and 300K to powder diffraction patterns calculated from the candidate models using the program VESTA [10]. We plot the data over a 2θ range where the T′ and T_{d} models have Bragg peaks that are well resolved from one another to highlight distinguishable features that differ between the phases in the measured XRD pattern.

II. SUPPLEMENTARY REFERENCES

[1] Lu, Pengchao et. al., Origin of the superconductivity of WTe_2 under pressure. Phys. Rev. B **94**, 224512 (2016).

[2] Kieffer, J. and Karkoulis, D. *PyFAI*, a versatile library for azimuthal regrouping. Journal of Physics: Conference Series **425**, 202012 (2013).

[3] Egami, T. and Billinge, S.J.L., Underneath the Bragg peaks: structural analysis of complex materials. *Elsevier* 2003 16.

[4] Petkov, V., Billinge S.J.L., Heising, J., and Kanatzidis M.G., Application of Atomic Pair Distribution Function Analysis to Materials with Intrinsic Disorder. Three-Dimensional Structure of Exfoliated-Restacked WS_2: Not Just a Random Turbostratic Assembly of Layers. Journal of the American Chemical Society **122**, 11571-11576 (2000).

[5] Juhas, P., Davis, T., Farrow, C.L., and Billinge, S.J.L., PDFgetX3: a rapid and highly automatable program for processing powder diffraction data into total scattering pair distribution functions. Journal of Applied Crystallography **46**, 560-566 (2013).

[6] Yang, X. et. al., xPDFsuite: an end-to-end software solution for high throughput pair distribution function transformation, visualization and analysis. Preprint at http://arxiv.org/abs/arXiv:1402.3163 (2014).

[7] Farrow, C. L. et. al., PDFfit2 and PDFgui: computer programs for studying nanostructure in crystals. Journal of Physics: Condensed Matter **19**, 335219 (2007).

[8] Brown, B. E. The crystal structures of WTe_2 and high-temperature $MoTe_2$. Acta Crystallographica **20**, 268-274 (1966).

[9] Puotinen, D. and Newnham, R. E., The crystal structure of $MoTe_2$. Acta Crystallographica **14**, 691-692 (1961).

[10] Momma, K. and Izumi, F., VESTA: a three-dimensional visualization system for electronic and structural analysis. Journal of Applied Crystallography **41**, 653-658 (2008).
